# From Pattern Recognition to Decision Guidance: Artificial Intelligence in Interstitial Lung Disease Diagnosis and Surveillance

**DOI:** 10.1002/resp.70298

**Published:** 2026-08-12

**Authors:** Deliang Yang, Xue Li, David Chi Leung Lam

**Affiliations:** ^1^ Department of Medicine, School of Clinical Medicine, Li Ka Shing Faculty of Medicine The University of Hong Kong Hong Kong SAR China; ^2^ The University of Hong Kong–Shenzhen Hospital China; ^3^ Centre for Safe Medication Practice and Research, Department of Pharmacology and Pharmacy, Li Ka Shing Faculty of Medicine The University of Hong Kong Hong Kong SAR China

**Keywords:** artificial intelligence, diagnosis, interstitial lung disease, multimodal decision‐support system, surveillance

## Abstract

- Current ILD guidelines centre diagnosis on clinical context, exposure and autoimmune assessment, high‐resolution CT, pulmonary function testing, and multidisciplinary discussion, while explicit endorsement of artificial intelligence remains limited.
- Some artificial intelligence tools have already entered ILD‐related clinical workflows by flagging interstitial abnormalities on routine CT, supporting non‐invasive assessment of suspected fibrotic ILD, and quantifying fibrotic burden and interval progression on serial imaging.
- Research‐stage AI models are beginning to address unresolved gaps in clinical guidelines by reducing diagnostic uncertainty in borderline cases, enabling subgroup‐specific risk stratification in connective tissue disease‐associated ILD, and forecasting progression or follow‐up needs from longitudinal multimodal data.
- The remaining need is to develop a validated personalised multimodal decision‐support system that can integrate imaging, clinical, serological, pathological, and longitudinal data to guide biopsy, surveillance, and management decisions for uncertain ILD cases.

Interstitial lung diseases (ILD) are a heterogeneous group of fibrotic and inflammatory parenchymal lung disorders that include idiopathic interstitial pneumonias, connective tissue disease (CTD)‐associated ILD [CTD‐ILD], hypersensitivity pneumonitis, and other ILDs caused by environmental or occupational exposures, or by medications and treatments [1]. Across different guidelines and routine clinical practice, diagnosis mainly relies on integrating clinical features, exposure history, autoimmune assessment, high‐resolution computed tomography (CT) of thorax, pulmonary function testing, and multidisciplinary discussion, with biopsy reserved for selected indeterminate cases [1, 2, 3, 4, 5]. Within different diagnostic pathways, explicit guideline endorsement of artificial intelligence (AI) remains limited.

The joint ATS/ERS/JRS/ALAT guidelines on idiopathic pulmonary fibrosis and progressive pulmonary fibrosis considered quantitative CT approaches based on machine learning and deep learning as promising but not yet ready for widespread clinical use without further validation and standardisation [3]. The ERS/EULAR guideline on CTD‐ILD just listed automated high‐resolution CT (HRCT) quantification and artificial intelligence‐based disease trajectory prediction as promising research priorities [5]. The ERS/ATS classification statement 2025 positioned AI within the interpretation of existing diagnostic tools and emphasised integration with clinical and laboratory data, multidisciplinary diagnosis, and explicit diagnostic confidence [1]. Thus, AI tools remain not yet guideline‐embedded for broad clinical application or routine decision‐making across diverse real‐world settings.

AI models have been proposed for implementation as software systems layered onto HRCT infrastructure used for supporting clinical diagnosis of ILD. These AI tools are trained on large CT image datasets and are designed to recognise radiological abnormalities consistently. Their roles differ across screening, pattern recognition, segmentation, and prognostic biomarker generation, as summarised in Table 1. Screening tools such as ScreenDx [6] flag CT scans with findings compatible with incidental pulmonary fibrosis, supporting case finding and referral. Pattern‐recognition tools, such as IQ‐UIP [7] and Fibresolve [8], move closer to diagnostic support by identifying usual interstitial pneumonia (UIP)‐like CT patterns or assisting idiopathic pulmonary fibrosis (IPF)‐related assessment in selected suspected ILD populations. Quantitative imaging‐biomarker tools such as e‐Lung, Lung Texture Analysis and Qureight measure or segment CT‐derived features, including reticulovascular abnormality, ground‐glass opacity, reticulation, honeycombing, lung volume, airway, vasculature and fibrosis [9, 10, 11, 12, 13]. Overall, these tools standardise CT assessment and improve workflow, but their common feature remains CT‐centred adjunctive support rather than replacement of multidisciplinary diagnosis.

**TABLE 1:** AI models that have been incorporated into CT reading workflows for ILD diagnosis, and the corresponding multimodal status.

Software	Main functions	Multimodal status
Fibresolve	UIP pattern recognition [8]; Prediction of mortality risk in ILD [14]	Embedding PFTs, age, and sex, allowing mortality risk stratification across ILD cohorts [14]
ScreenDx	Automated CT screening for identifying incidental pulmonary fibrosis [6]	Not multimodal; CT scans only
IQ‐UIP	UIP classification on chest CT [7].	Not multimodal; CT scans only
e‐lung	Quantifying thoracic CT biomarkers in ILD, and deriving outputs include the weighted reticulovascular score (WRVS) and biomarkers [10].	Not multimodal; CT scans only
Lung Texture Analysis	Automated CT pattern quantification of Ground‐Glass Opacity (GGO), reticulation, honeycombing [9].	Not multimodal; CT scans only
Qureight	Quantitative HRCT segmentation platform in IPF, including four anatomical deep‐learning CT biomarkers around segmentation of lung, airways, vasculature, and fibrosis [12].	Not multimodal; CT scans only

*Note:* Multimodal status refers to whether the model incorporates non‐imaging clinical variables in addition to CT data.

Abbreviations: AI, artificial intelligence; CT, computed tomography; GGO, ground‐glass opacity; HRCT, high‐resolution computed tomography; ILD, interstitial lung disease; IPF, idiopathic pulmonary fibrosis; PFTs, pulmonary function tests; UIP, usual interstitial pneumonia; WRVS, weighted reticulovascular score.

Because CTD‐ILD is an ILD subtype embedded within systemic autoimmune disease, diagnosis and baseline risk assessment of CTD‐ILD should be framed as a multisource pathway that integrates HRCT with clinical phenotype, pulmonary function, inflammatory markers, exercise assessment and disease‐specific context [5]. This highlights the distance between clinical CTD‐ILD assessment and currently available CT‐workflow tools (Table 1), because they still mainly operate on CT images rather than on the full diagnostic context. None of them can by itself attribute ILD cause, decide on biopsy, or personalise management across CTD‐ILD. Fibresolve has moved furthest by incorporating pulmonary function tests (PFTs), age and sex [14], but it remains a prognostic tool rather than a full clinical diagnostic and decision‐support system.

On the other hand, several AI models have been formulated to target unresolved gaps highlighted by current ILD guidelines, including algorithmic attempts to resolve diagnostic uncertainty in borderline cases [1, 15, 16, 17], subgroup‐specific risk stratification in CTD‐ILD [5, 18, 19, 20], and personalised follow‐up planning before overt physiological decline [1, 5, 21, 22, 23]. For diagnostic uncertainty, deep‐learning classifiers, such as SOFIA [24] and related HRCT‐based models, have shown that continuous UIP probability scoring can reduce inter‐observer variability and support borderline cases on multidisciplinary discussion. Systems such as PMFF‐Net and deep‐learning content‐based image retrieval extend this approach beyond UIP towards NSIP and organising pneumonia, trying to resolve the ambiguity that may arise when there exists no absolute diagnostic pathognomonic test [15, 17].

In CTD‐ILD, AI models have been shown to be able to quantify systemic sclerosis (SS)‐ILD disease burden on CT, forecasting lung function decline in systemic SS‐ILD, and predicting rapid progression in idiopathic inflammatory myopathy‐associated ILD [18, 19, 20]. Likewise, multimodal longitudinal systems, such as Mei's integrated diagnosis‐prognosis model [21] and progression‐focused pulmonary fibrosis models like Fibrosis‐Net [22] and Fibro‐CoSANet [23] begin to address personalised surveillance and progression forecasting before overt physiological decline by using combinations of CT, spirometry, and clinical metadata to predict future ILD behaviour. These studies suggest a shift from classification based on individual radiological patterns towards subgroup‐specific diagnosis and longitudinal support for disease follow‐up or progression.

Despite the increasing availability of new AI tools as above, an obvious unmet need across current ILD guidelines is the lack of a validated personalised multimodal decision‐support system that can combine imaging, clinical, serological, pathological, and longitudinal data to enhance diagnostic confidence or to guide biopsy, or disease surveillance and management decisions in indeterminate clinical situations [1, 2, 5]. Afterall, current clinical ILD modelling remains at radiological pattern classification, subgroup risk prediction, or progression forecasting. This kind of clinical modelling lacks the ability to inform whether invasive testing can be avoided or follow‐up should be adjusted for in individual patients [15, 16, 17, 18, 19, 21, 22, 23, 25]. In other medical specialty fields, especially oncology, multimodal AI systems already illustrate this next step by combining radiology, pathology, genomics, and clinical variables to support clinical decisions in individual patients [25, 26]. Comparable systems should be developed for the specific characteristics or co‐morbidities of ILD and even tailored to different subtypes, but reaching that point will require harmonised longitudinal datasets, multimodal integration across specialties, explainable uncertainty‐aware models, and prospective validation within real‐world multidisciplinary workflows [27, 28].

## Author Contributions

**Deliang Yang:** conceptualization (lead), investigation (lead), writing – original draft (lead). **Xue Li:** conceptualization (lead), supervision (lead), writing – review and editing (lead). **David Chi Leung Lam:** writing – review and editing (supporting).

## Funding

The authors have nothing to report.

## Conflicts of Interest

X.L. received research grants or contracts from the Health and Medical Research Fund (HMRF) Main Scheme and HMRF Fellowship Scheme of the Hong Kong Special Administrative Region (SAR), China, and from the Research Grants Council Early Career Scheme, Research Impact Fund (Hong Kong SAR); was former nonexecutive director of Advanced Data Analytics for Medical Science Limited, Hong Kong SAR, China; received commission grants from Department of Health, Government of Hong Kong SAR, China; commission grants from Hospital Authority of Hong Kong SAR, China, internal funding from the University of Hong Kong, Hong Kong SAR, China, and research or education grants from Pfizer, Janssen and Bristol Myers Squibb (BMS), Novartis; received consultancy fees from Merck Sharp & Dohme, Pfizer, Open Health and The Office of Health Economics; and received honoraria for associate editorship from Nature Springer. X.L. is an Editorial Board member of *Respirology* and D.C.L.L. is Co‐Editor in Chief of *Respirology*—both were excluded from all editorial decision‐making related to this article.
